# Too Many Appointments: Assessing Provider and Nursing Perception of Barriers to Referral for Outpatient Palliative Care

**DOI:** 10.1089/pmr.2020.0114

**Published:** 2021-05-17

**Authors:** Julia L. Agne, Erin M. Bertino, Madison Grogan, Jason Benedict, Sarah Janse, Michelle Naughton, Christine Eastep, Michael Callahan, Carolyn J. Presley

**Affiliations:** ^1^ Division of Palliative Medicine, The Ohio State University Wexner Medical Center, Columbus, Ohio, USA.; ^2^ Division of Medical Oncology, The Ohio State University James Comprehensive Cancer Center, Columbus, Ohio, USA.; ^3^ Center for Biostatistics, The Ohio State University Wexner Medical Center, Columbus, Ohio, USA.; ^4^ Cancer Control and Prevention, The Ohio State University James Comprehensive Cancer Center, Columbus, Ohio, USA.; ^5^ Department of Oncology Nursing, The Ohio State University James Comprehensive Cancer Center, Columbus, Ohio, USA.

**Keywords:** barrier, cancer, outpatient, palliative care, referral, time cost

## Abstract

***Background:*** Integration of early outpatient palliative care for patients with advanced cancer requires overcoming logistical constraints as well as attitudinal barriers of referring providers. This pilot study assessed provider perception of logistical and attitudinal barriers to outpatient palliative care referral as well as provider acceptability of an embedded onco-palliative clinic model.

***Methods:*** This was a cross-sectional survey-based study of medical oncologists, palliative care physicians, advanced practice providers (APP), and oncology nurses at a large U.S. academic center. Participants were invited to participate through anonymous online survey. Participants rank ordered logistical barriers influencing referral to an outpatient palliative clinic. Respondents indicated level of agreement with attitudinal perception of palliative care and acceptability of an embedded palliative clinic model through five-item Likert-like scales.

***Results:*** There were a total of 54 study participants (28 oncology physicians/APPs, 15 palliative physicians/APPs, and 11 oncology nurses). Across the three cohorts, most survey respondents ranked “time burden to patients” as the primary logistical barrier to outpatient palliative care referral. Both oncology and palliative providers indicated comfort with primary palliative care skills although palliative providers were more comfortable with symptom management compared with oncology providers (93.3% vs. 32.2%). A majority of participants (94.9%) were willing to refer to a palliative care provider embedded within an oncology clinic.

***Conclusion:*** Additional health care time cost to patients is a major barrier to outpatient palliative care referral. Embedding a palliative care provider in an oncology clinic may be an acceptable model to increase patient access to outpatient palliative care while supporting the oncology team.

## Introduction

Patients with advanced cancer, particularly lung cancer, benefit from early integration of palliative care with standard oncology care.^1–4^ The American Society of Clinical Oncology (ASCO) recommends that all patients with advanced cancer receive outpatient palliative care early in their disease course concurrent with active treatment.^5^ Barriers to palliative care referrals previously described include limited resource availability, financial constraints, time burden of additional clinic appointments, patient and caregiver fatigue, and negative perception of end-of-life care by referring providers.^6,7^ Successful palliative care referral requires overcoming attitudinal barriers as well as logistical limitations in palliative care delivery as perceived by the referring provider.^8–10^

In considering palliative care referral, oncology providers frequently weigh a patient's prognosis and unmet palliative needs with limited availability of subspecialty palliative care resources.^11^ For the past decade, palliative care programs at National Cancer Institute (NCI)-designated cancer centers have experienced significant growth in outpatient palliative clinics, outpacing inpatient palliative or hospice services.^12^ Expansion of outpatient palliative services has led to significant variability in referral criteria, timing of referral, and models of outpatient palliative care delivery across cancer centers worldwide.^13–17^

Prior survey-based studies have offered insight into oncologists' perception of palliative care services,^9,10^ yet none have attempted to ascertain both attitudinal barriers as well as logistical barriers affecting oncologists' decision in referring patients for outpatient palliative care. In addition, the attitudes of oncology nursing staff as well as advanced practice providers (APPs, nurse practitioners, and physician assistants) toward palliative care services are unknown. With the growth of palliative clinics, oncology nurses are increasingly empowered to advocate for palliative care resources for patients with advanced cancer.^18–20^ Oncology nurses offer unique perspectives that may more closely reflect barriers faced by patients and their families when attempting to complete palliative care consultation.^20^

To address these questions, we conducted a cross-sectional survey-based study of physicians, APP, and oncology nurses to explore the perception of logistical and attitudinal barriers affecting referral to subspecialty palliative care. This pilot study also explores acceptability of an embedded palliative care clinic model as a means to overcome attitudinal and practical barriers to outpatient palliative care consultation.

## Methods

This cross-sectional study was part of a larger pilot project to assess the impact of embedding a palliative physician in a thoracic oncology clinic. At the time of this study, all participants interfaced with outpatient palliative care through a freestanding palliative clinic that is available to all cancer patients at this institution. We utilized an anonymous online survey of physicians, APP, and oncology clinic nurses in the Divisions of Medical Oncology and Palliative Medicine at the Ohio State University Comprehensive Cancer Center. APP included certified nurse practitioners and physician assistants. All physicians and APPs in medical oncology and palliative medicine were invited to participate through institutional listserv. For this exploratory pilot study, only nursing staff in the thoracic oncology clinic were invited to participate in the nursing cohort. Potential study participants (*n* = 130) received an institutional review board (IRB)-approved e-mail invitation that explained the nature of the study and included an online link to the survey with a consent form for study participation. Potential study participants received a reminder e-mail if they had not completed the study survey within two weeks of receiving the initial study invitation. The study was approved by the IRB.

### Survey development

The study survey was a 29-item questionnaire exploring factors influencing provider referral to outpatient palliative care services (Supplementary Appendix SA1). The survey comprised three main sections—logistical barriers to outpatient palliative care referral, attitudes toward palliative care, and acceptability of an embedded palliative care clinic model. The survey also included questions about professional training level, years of postgraduate work experience, and amount of palliative care training. Survey items pertaining to logistical barriers and acceptability of an embedded palliative clinic model were developed through literature review and multidisciplinary team consensus, whereas survey items pertaining to attitudinal perception of palliative care were reproduced from previously published surveys with permission from investigators at MD Anderson Cancer Center.^9,10^

Logistical barriers to outpatient palliative referral were elucidated through literature review and consensus of a multidisciplinary stakeholder committee, comprising two medical oncologists, a palliative care physician, and two nursing administrators. The committee identified five main logistical barriers to outpatient palliative care referral at this institution; additional health care cost to patient, remote clinic location, time burden of additional appointments, perceived lack of added value to patient care, and patient refusal of palliative care referral. Study participants were asked to rank these barriers from “1” (primary barrier) to “5” (least important barrier) when deciding on whether to refer a patient for outpatient palliative care services. As this was a pilot study, this was the first utilization of these questions.

Survey items regarding provider attitudes toward patient characteristics warranting palliative care referral, perception of “palliative care” service name, level of comfort with primary palliative care skills, and degree of clinician support were reproduced from previously published surveys with permission from investigators at MD Anderson Cancer Center.^9,10^ These survey items were previously pilot tested with hematology and medical oncology providers (physicians and APPs) at a single large academic cancer center although reliability and validity of these survey items were not previously reported.^9,10^

To assess acceptability of an embedded palliative clinic model, participants indicated their level of agreement with the statement “I would refer patients to a palliative care provider working in my outpatient clinic” through five-item Likert-like scale (“strongly agree,” “agree,” “neither agree nor disagree,” “disagree,” and “strongly disagree”).

### Data analysis

Descriptive statistics were used to summarize the data by provider type (oncology, palliative, or nursing). Fisher's exact test was used to test for differences between provider types for those who chose “strongly agree” versus all other options. As the questions addressing level of comfort of care among providers measured similar underlying factors, these questions were used to estimate the reliability of the instrument using Cronbach's alpha. These data are exploratory and only questions believed to be of interest before data collection began were tested for statistical significance. All analyses and graphs were generated in R version 4.0.

## Results

### Response rate

The overall response rate for all providers was 41.5% (54/130). In the Division of Medical Oncology, overall survey response rate was 30.8% (28/91), which reflects participation from medical oncologists (19/65; 29.2%) and APP (9/26; 34.6%). In the Palliative Medicine division, overall survey response rate was 53.6% (15/28), which reflects participation from palliative physicians (9/15; 60.0%) and APP (6/13; 46.1%). All thoracic oncology clinic nurses (11/11; 100%) responded to this survey.

### Participant characteristics

The characteristics of study participants are provided in Table 1. There was a comparable level of postgraduate experience between respondents in medical oncology and palliative care. A majority of oncology providers (21/28; 75%) reported at least some level of palliative care education—palliative clinical rotation (9/28; 32%) or palliative care lectures (10/28; 36%). Oncology nurses reported little or no training in palliative care.

**Table 1.** Participant Demographics by Provider Type

Characteristic, *n* (%)	Medical oncology, *N* = 28	Nurses, *N* = 11	Palliative, *N* = 15
Provider training level			
Physician	19 (68)	N/A	9 (60)
APP	9 (32)	N/A	6 (40)
Postgraduate experience			
<10 Years	16 (57)	6 (55)	8 (53)
≥10 Years	12 (43)	4 (36)	7 (47)
Unknown	0 (0)	1 (9)	0 (0)
Palliative care training^a^			
Formal palliative care fellowship (one year or more)	2 (7)	N/A	9 (60)
Formal palliative care rotation (one month or more)	9 (32)	N/A	2 (13)
Palliative care courses, continuing medical education lectures or conferences	10 (36)	5 (45)	4 (27)
No training	7 (25)	6 (55)	N/A

^a^ At this institution, nurses do not have the opportunity to undergo a formal palliative care fellowship or rotation.

APP, advanced practice providers.

### Logistical barriers to palliative care referral

**FIG. 1.**
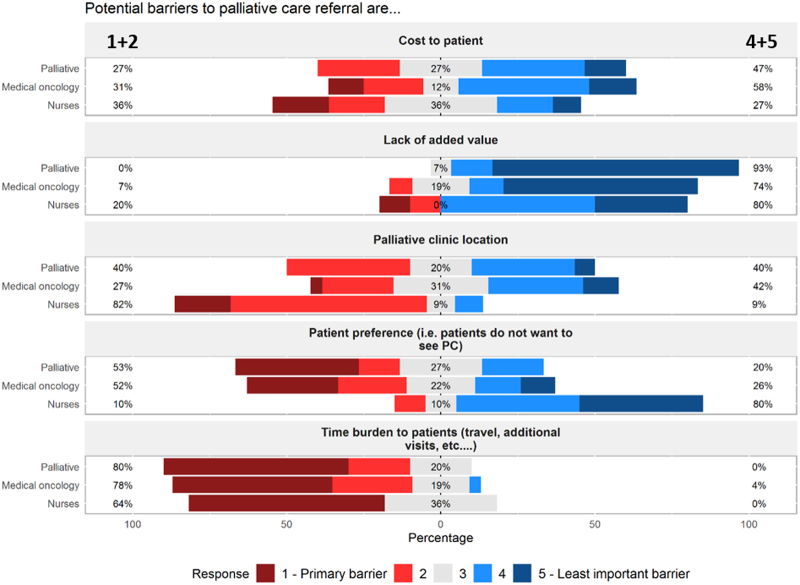
Logistical barriers to outpatient palliative care referral. Logistical barriers to outpatient palliative care referral as ranked by medical oncology providers, palliative care providers, and oncology nursing staff. Left-sided percentages represent cumulative percentage of ranked order first and second most important barrier as designated by survey participants. Gray bars represent percentage of respondents ranking an item as third most important barrier. Right-sided percentages represent cumulative percentage of ranked order fourth and fifth (least important) barrier to outpatient palliative care referral.

Figure 1 summarizes the perception of logistical barriers to outpatient palliative care referral as ranked by medical oncology providers, palliative care providers, and oncology nursing staff. A majority of respondents in all three groups (medical oncology 78%, palliative 80%, and nurses 64%) ranked “time burden to patients” as either the primary or secondary barrier when considering referral to the outpatient palliative care for cancer patients. Clinicians in both medical oncology and palliative care ranked “patient preference” as an influential logistical barrier to palliative clinic referral with more than half of respondents (medical oncology 52% and palliative 53%) ranking this as either the first or second most important barrier. Most oncology nurses (82%) ranked “palliative clinic location” as the primary or secondary barrier to outpatient palliative care, whereas oncology and palliative clinicians were more equivocal on this barrier. A majority of respondents ranked “lack of added value” as the least influential barrier.

### Provider perception of palliative care skills

**FIG. 2.**
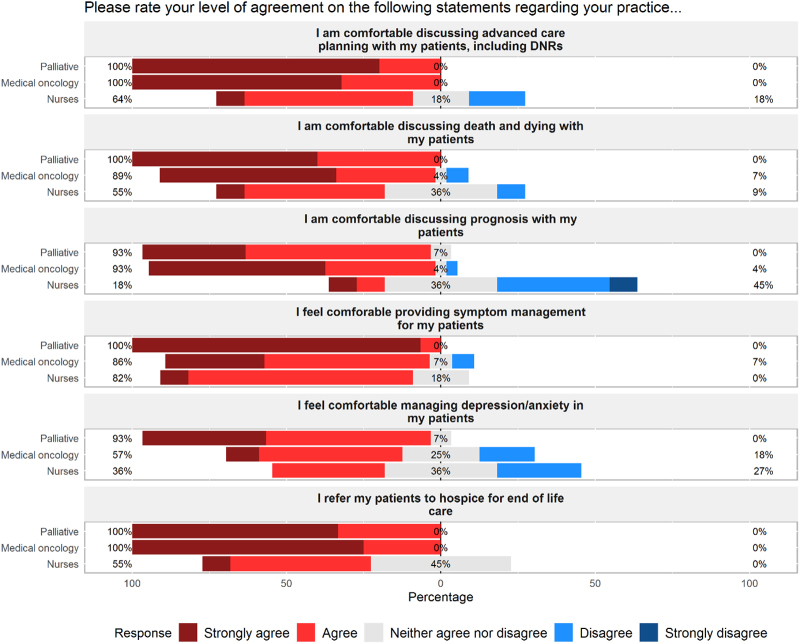
Provider perception of palliative care skills. Primary palliative care skills as perceived by medical oncology providers, palliative care providers, and oncology nursing staff. Left-sided percentages represent cumulative percentage of “strongly agree” and “agree” responses as designated by survey participants. Gray bars represent percentage of respondents providing neutral response (“neither agree nor disagree”). Right-sided percentages represent cumulative percentage of “disagree” and “strongly disagree” responses.

As summarized in Figure 2, clinicians in both medical oncology and palliative care reported feeling comfortable discussing advance care planning, death and dying, and prognosis with patients. All palliative care clinicians reported feeling comfortable providing symptom management (93.3% “strongly agree”), whereas oncology providers indicated less comfort with symptom management (32.1% “strongly agree”) (Fisher's exact test: *p* < 0.01). Oncology providers also reported feeling less comfortable with managing mood symptoms such as anxiety and depression (10.7% “strongly agree”) compared with palliative care providers (40% “strongly agree”) (Fisher's exact test: *p* < 0.05). All physicians and APPs indicated that they agree or strongly agree with the statement “I refer my patients to hospice for end of life care.” Among the five questions addressing comfort of care, Cronbach's alpha was estimated to be 0.80 (95% confidence interval: 0.71–0.88) indicating acceptable to good reliability.

### Provider perception of support

**FIG. 3.**
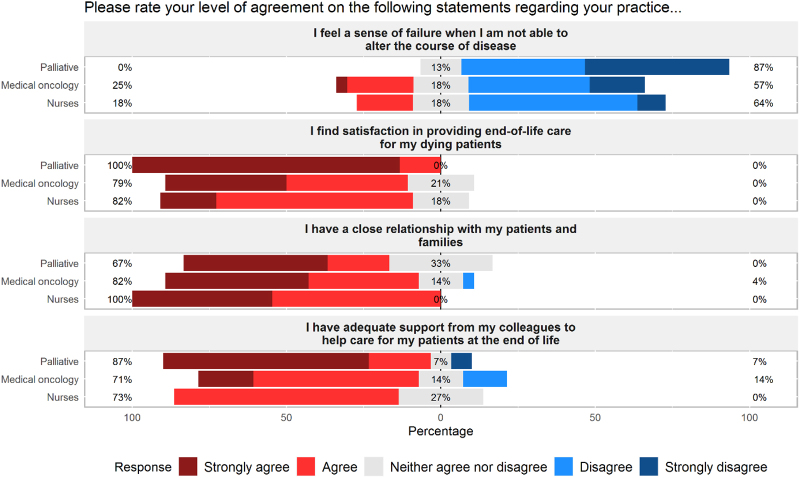
Provider perception of support. Professional support as perceived by medical oncology providers, palliative care providers, and oncology nursing staff. Left-sided percentages represent cumulative percentage of “strongly agree” and “agree” responses as designated by survey participants. Gray bars represent percentage of respondents providing neutral response (“neither agree nor disagree”). Right-sided percentages represent cumulative percentage of “disagree” and “strongly disagree” responses.

Figure 3 reflects responses to survey items on support and job satisfaction when caring for patients with a terminal prognosis. A subset of medical oncology providers (25%) and oncology nurses (18%) indicated that they feel a sense of failure when unable to alter a patient's disease course, whereas no palliative care respondents agreed with this statement. Although all palliative participants reported feeling satisfaction in providing end-of-life care (86.7% “strongly agree” and 13.3% “agree”), a subset of medical oncology (21%) and nursing providers (18%) responded neutrally to this item. Oncology participants (physicians/APPs 82% and nurses 100%) reported having close relationships with patients and families, whereas the palliative cohort responded more neutrally to this statement (33% “neither agree nor disagree”). Palliative providers endorsed the strongest support from colleagues in caring for patients at the end of life (66.7% “strongly agree” and 20% “agree”), whereas perception of clinician support was felt less strongly among medical oncology providers (17.9% “strongly agree” and 53.6% “agree”) and oncology nurses (0% “strongly agree” and 72.7% “agree”).

### Acceptability of an embedded palliative clinic model

Most medical oncology providers and all oncology nurses indicated that they would be willing to refer patients to a palliative provider working within an outpatient oncology clinic (Table 2). Only two (7.1%) medical oncology providers responded neutrally to this statement.

**Table 2.** Acceptability of Embedded Outpatient Palliative Care Model

I would refer patients to a Palliative Care provider working in my outpatient clinic, *n* (%)^a^	Medical oncology providers, *N* = 28	Nursing staff, *N* = 11
Strongly agree	17 (61)	8 (73)
Agree	9 (32)	3 (27)
Neither agree nor disagree	2 (7)	0 (0)
Disagree	0 (0)	0 (0)
Strongly disagree	0 (0)	0 (0)

^a^ Palliative care providers were excluded from this survey item.

### Patient characteristics for palliative care referral and perception of palliative care service name

Participants across all three cohorts largely agreed with palliative care referral for patients newly diagnosed with cancer, no longer receiving cancer treatment for advanced disease, receiving cancer treatment with palliative intent, and actively receiving treatment with curative intent (Supplementary Fig. S1). Most participants across all three cohorts disagreed that the service name “Palliative Care” is a barrier to referral, synonymous with end-of-life care, or decreases hope in patients and families (Supplementary Fig. S2). Unlike medical oncology and palliative providers, a majority of oncology nurses (64%) indicated that the name “Palliative Care” is associated with treatment of chemotherapy side effects (Supplementary Fig. S2).

## Discussion

This exploratory cross-sectional study highlights practical barriers to integration of early palliative care with standard oncology care as recommended by current ASCO guidelines.^5^ As part of a larger pilot project to embed a palliative care provider in a thoracic oncology clinic, this survey-based study explored logistical and attitudinal barriers influencing referral to a freestanding palliative clinic before opening of an embedded onco-palliative clinic model. At this institution, medical oncology providers and inpatient palliative consult teams are the primary gatekeepers to outpatient palliative clinic referral. By capturing perceptions of the overall referral base, this study explores perceived barriers in relation to a freestanding outpatient palliative clinic before piloting embedded palliative providers in oncology clinics. To assess nursing perception of outpatient palliative care, thoracic oncology nurses were included in this study to elicit baseline perspective in a targeted nursing cohort before embedding a palliative provider in the thoracic oncology clinic. Eliciting the perspective of referring providers and nursing staff was pertinent in exploring barriers to palliative care referral, acceptance of a new embedded palliative clinic model, and providing baseline data to explore how perceptions and barriers change with embedded outpatient onco-palliative care.

By ranking logistical barriers to outpatient palliative care referral in this study, both oncology and palliative care providers recognize the significant time burden of standard oncology care for patients with advanced cancer. Although outpatient palliative care services have expanded at many NCI-designated cancer centers for the past decade, most palliative clinics operate in a freestanding model that is separate from a patient's outpatient oncology clinic.^12^ At the time of this survey, patient access to outpatient palliative care was only available through a freestanding palliative clinic operating separately from outpatient oncology care at this institution. Providing embedded palliative care within an oncology clinic may facilitate earlier palliative care referral while decreasing health care time cost to patients. Across diverse cancer diagnoses, Yabroff et al. described a significant increase in health care-related time cost to patients in the last year of life when compared with the first year after cancer diagnosis.^21^ Perception of additional health care time cost, particularly in patients approaching the end of life, amplifies reluctance of providers to recommend and patients to accept referral to nonembedded palliative care services. For these reasons, early outpatient palliative care referral (>12 months before death) is more likely to occur for patients with longer disease course or to address treatment-related symptoms rather than longitudinal palliative care management across the disease continuum.^22,23^ Embedding a palliative care provider within an oncology clinic may decrease the perception of additional time cost to patients typically experienced by a freestanding palliative clinic model. Time cost to patients is further reduced when an embedded palliative provider has flexibility to see patients concurrently with the oncology team or during infusion of systemic cancer treatment.

Similar to Hui et al.,^9^ this study demonstrated a high comfort level with primary palliative care skills such as prognostication, advance care planning, and cancer symptom management among oncology providers. By surveying palliative care providers, this study is unique in that palliative care participants indicated a higher level of comfort with emotional and physical symptom management compared with their oncology colleagues. This finding reflects subspecialty training of palliative providers in complex symptom management, whereas oncology training focuses on treatment of the underlying malignancy. In addition, palliative care providers reported less comfort with prognostication compared with oncology providers, which may reflect less knowledge of newer cancer treatments as perceived by palliative care providers. These findings highlight the need for close collaboration between these two specialties to facilitate end-of-life decision making in patients with advanced cancer. An embedded onco-palliative clinic model may facilitate closer collaboration between medical oncologists and palliative providers by facilitating shared goals of care discussions and symptom assessments in the oncology clinic.

Although respondents frequently agreed on survey items, differences between medical oncology and palliative providers were often most interesting in the strength of their responses. For example, when asked about their comfort level when providing symptom management to patients, both specialists indicated a high level of comfort (100% vs. 86% “agree” or “strongly agree” palliative care vs. medical oncology). Palliative providers, however, appeared substantially more comfortable with symptom management in that they more frequently indicated a strong comfort level versus medical oncologists (“strongly agree” 93% vs. 32%, respectively). Although other studies chose to collapse levels of the Likert scale,^9,10^ doing so results in the loss of these subtle but important differences. Therefore, we chose not to collapse the Likert scale levels in this study.

Medical oncologists with access to on-site palliative care report higher overall job satisfaction, which is clinically relevant as oncologists with low job satisfaction are less likely to discuss prognosis with their patients.^24^ In addition to supporting patient care, palliative care providers work closely with oncology teams to process the emotional toll of caring for dying patients. Although both oncology and palliative providers in this study cited satisfaction in providing end-of-life care, a subset of oncology providers expressed a sense of failure when unable to alter the disease course of a dying patient, which was not reported by palliative care providers. This finding may reflect the focus of disease curability in medical oncology, whereas patient death is widely accepted in palliative care. Feelings of failure may also reflect a sense of closeness oncology providers and nurses develop with their patients, which results in both personal satisfaction and emotional burden of transitioning patients to end-of-life care.

Although this pilot study yielded useful and interesting data to guide development of an embedded onco-palliative clinic model, the study had several limitations. This study was limited by low overall response rates among both oncology and palliative providers. As participation was voluntary and not compensated, low response rate may be reflective of the lack of financial incentive for survey completion. This study also reflects only the perception of clinicians and nurses at a single academic cancer center with on-site palliative care. Logistical barriers to palliative care integration in this study may not be reflective of other community oncology practices. Among nursing staff, only thoracic oncology clinic nurses were included in this study population. Further research is recommended to elucidate the larger nursing perspective pertaining to outpatient palliative care. Potential barriers to an embedded palliative clinic model was beyond the scope of this survey-based study. The study was not powered to detect distinct differences in provider types and was exploratory in nature. Further research is needed to confirm the findings of this study.

## Conclusion

The goal of this study was to explore perceptions of barriers to outpatient palliative care referral at a large academic cancer center. Time cost to patients was identified as the primary logistical barrier to outpatient palliative care consultation. Although resource intense, the concept of embedding a palliative care provider in an oncology clinic to reduce health care time cost to patients was largely acceptable to both oncology providers and nursing staff. This study also suggests that closer collaboration between medical oncology and palliative care providers may improve comfort with prognostication while providing emotional support for the medical team in caring for dying patients.

## Supplementary Material

Supplemental data

Supplemental data

Supplemental data

## Supplementary Material

Supplementary Figure S2

Supplementary Appendix SAP1

## Abbreviations Used

APP: advanced practice providers
ASCO: American Society of Clinical Oncology
IRB: institutional review board
NCI: National Cancer Institute
