# Supplementary material for: Too Many Appointments: Assessing Provider and Nursing Perception of Barriers to Referral for Outpatient Palliative Care
Source: Palliat Med Rep. 2021 May 17;2(1):137–45. doi: 10.1089/pmr.2020.0114 (PMC8241388; doi:10.1089/pmr.2020.0114)

**Supplemental Figure 2:** Perception of Palliative Care Service Name

Palliative care service name as perceived by medical oncology providers, palliative care providers, and oncology nursing staff. Left-sided percentages represent cumulative percentage of “strongly agree” and “agree” responses as designated by survey participants. Grey bars represent percentage of respondents providing neutral response (“neither agree nor disagree”). Right-sided percentages represent cumulative percentage of “disagree” and “strongly disagree” responses.


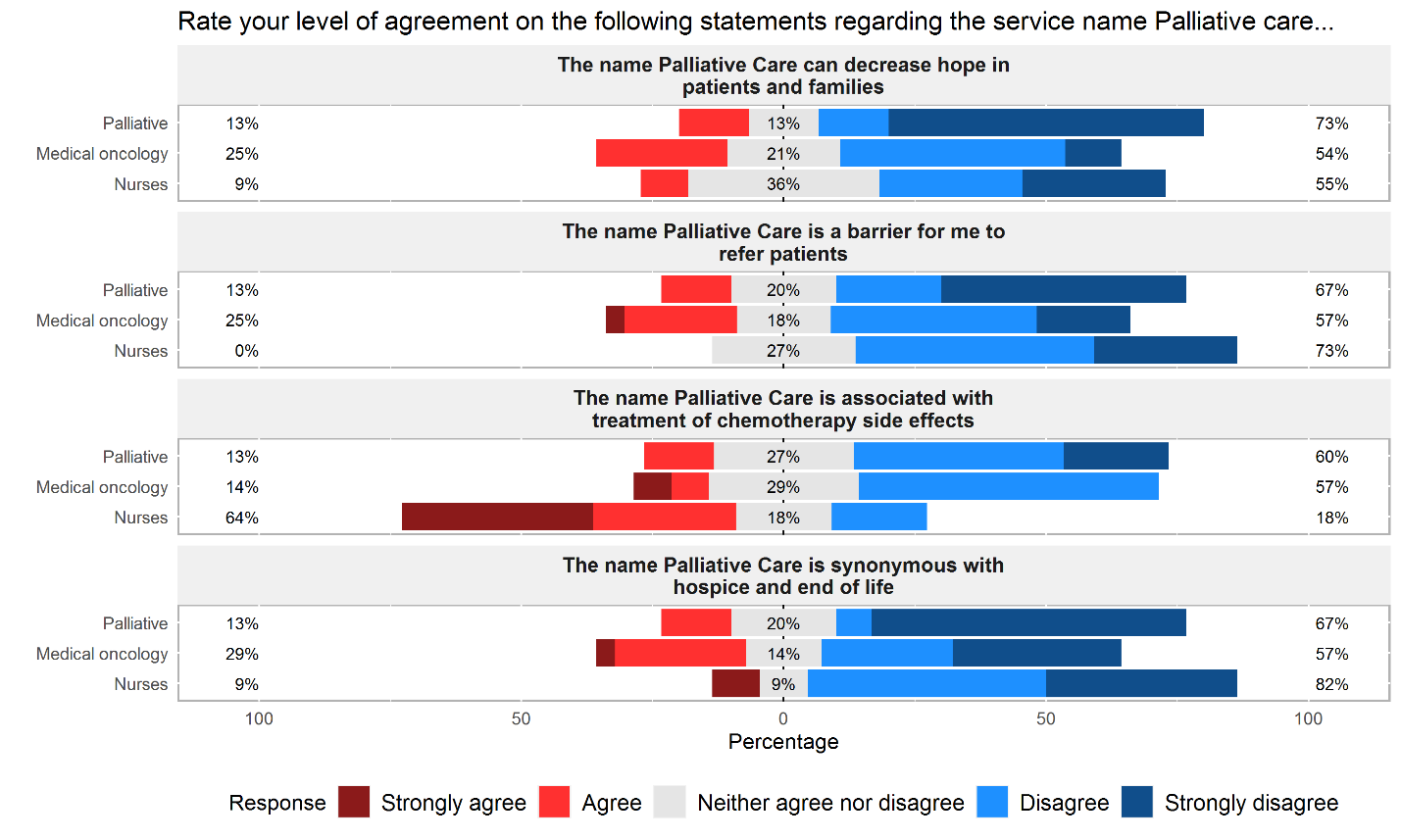

Supplement: Supplemental data [file Supp_FigS2.docx]
