# Supplementary material for: Too Many Appointments: Assessing Provider and Nursing Perception of Barriers to Referral for Outpatient Palliative Care
Source: Palliat Med Rep. 2021 May 17;2(1):137–45. doi: 10.1089/pmr.2020.0114 (PMC8241388; doi:10.1089/pmr.2020.0114)
Supplement: Supplemental data [file Supp_Data.pdf]

Osu Provider Survey Attitudes And Beliefs About Palliative Care

Please complete the survey below.

Thank you!

Explanation: We have designed a pilot study to evaluate embedding a palliative care physician in medical oncology clinic. The palliative care physician would be available to see patients in conjunction with their medical oncology provider for palliative care assessment and ongoing outpatient management. This survey is to evaluate current provider beliefs and practices.

Your responses are anonymous. We will plan to repeat this survey three times so consistent participation is appreciated.

Please rate your level of agreement on the following statements:

I would use the Palliative Care service for a patient with symptoms and who is:

1) Newly diagnosed with cancer

- ◯ Strongly Agree
- ◯ Agree
- ◯ Neither Agree nor Disagree
- ◯ Disagree
- ◯ Strongly Disagree

2) Undergoing active primary treatment for cancer (curative intent)

- ◯ Strongly Agree
- ◯ Agree
- ◯ Neither Agree nor Disagree
- ◯ Disagree
- ◯ Strongly Disagree

3) Without evidence of active cancer

- ◯ Strongly Agree
- ◯ Agree
- ◯ Neither Agree nor Disagree
- ◯ Disagree
- ◯ Strongly Disagree

4) Receiving active treatment for advanced cancer (palliative intent)

- ◯ Strongly Agree
- ◯ Agree
- ◯ Neither Agree nor Disagree
- ◯ Disagree
- ◯ Strongly Disagree

5) No longer receiving cancer treatment

- ◯ Strongly Agree
- ◯ Agree
- ◯ Neither Agree nor Disagree
- ◯ Disagree
- ◯ Strongly Disagree

6) I would refer patients to a Palliative Care provider working in my outpatient clinic

- ◯ Strongly Agree
- ◯ Agree
- ◯ Neither Agree nor Disagree
- ◯ Disagree
- ◯ Strongly Disagree

Please rate your level of agreement on the following statements regarding the service name "Palliative Care."

7) Service name is a barrier for me to refer patients
- ◯ Strongly Agree
- ◯ Agree
- ◯ Neither Agree nor Disagree
- ◯ Disagree
- ◯ Strongly Disagree

8) Service name is synonymous with hospice and end of life
- ◯ Strongly Agree
- ◯ Agree
- ◯ Neither Agree nor Disagree
- ◯ Disagree
- ◯ Strongly Disagree

9) Service name can decrease hope in patient and families
- ◯ Strongly Agree
- ◯ Agree
- ◯ Neither Agree nor Disagree
- ◯ Disagree
- ◯ Strongly Disagree

10) Service name is associated with treatment of chemotherapy side effects
- ◯ Strongly Agree
- ◯ Agree
- ◯ Neither Agree nor Disagree
- ◯ Disagree
- ◯ Strongly Disagree

Please rate your level of agreement on the following statements regarding your practice.

11) I am comfortable discussing advance care planning with my patients, including DNR orders.
- ◯ Strongly Agree
- ◯ Agree
- ◯ Neither Agree nor Disagree
- ◯ Disagree
- ◯ Strongly Disagree

12) I have a close relationship with my patients and families
- ◯ Strongly Agree
- ◯ Agree
- ◯ Neither Agree nor Disagree
- ◯ Disagree
- ◯ Strongly Disagree

13) I am comfortable discussing prognosis with my patients.
- ◯ Strongly Agree
- ◯ Agree
- ◯ Neither Agree nor Disagree
- ◯ Disagree
- ◯ Strongly Disagree

14) I refer my patients to hospice for end of life care.
- ◯ Strongly Agree
- ◯ Agree
- ◯ Neither Agree nor Disagree
- ◯ Disagree
- ◯ Strongly Disagree

15) I am comfortable discussing death and dying with my patients
- ◯ Strongly Agree
- ◯ Agree
- ◯ Neither Agree nor Disagree
- ◯ Disagree
- ◯ Strongly Disagree

16) I find satisfaction in providing end-of-life care for my dying patients.
- ◯ Strongly Agree
- ◯ Agree
- ◯ Neither Agree nor Disagree
- ◯ Disagree
- ◯ Strongly Disagree

17) I have adequate support from my colleagues to help care for my patients at the end of life.
- ◯ Strongly Agree
- ◯ Agree
- ◯ Neither Agree nor Disagree
- ◯ Disagree
- ◯ Strongly Disagree

18) I feel a sense of failure when I am not able to alter the course of disease.
- ◯ Strongly Agree
- ◯ Agree
- ◯ Neither Agree nor Disagree
- ◯ Disagree
- ◯ Strongly Disagree

19) I feel comforable providing symptom management for my patients.
- ◯ Strongly Agree
- ◯ Agree
- ◯ Neither Agree nor Disagree
- ◯ Disagree
- ◯ Strongly Disagree

20) I feel comfortable managing depression/anxiety in my patients.
- ◯ Strongly Agree
- ◯ Agree
- ◯ Neither Agree nor Disagree
- ◯ Disagree
- ◯ Strongly Disagree

Barriers to Palliative Care Referral
Please rank the following potential barriers to palliative care referral at Ohio State, with #1 being the primary barrier.

	1- primary barrier	2	3	4	5 - least important barrier
21) Cost to patient (visit co-pay, etc)	◯	◯	◯	◯	◯
22) Clinic location (parking, etc)	◯	◯	◯	◯	◯
23) Time burden to patients (travel, additional visits)	◯	◯	◯	◯	◯
24) Lack of added value	◯	◯	◯	◯	◯
25) Patient preference (i.e. patients do not want to see PC)	◯	◯	◯	◯	◯

Demographics

Please provide us with some basic information about yourself. All results will be reported anonymously.

26) I am a specialist in the following discipline:
- ◯ Medical oncology - Thoracic
- ◯ Medical oncology - Other
- ◯ Palliative Medicine

27) I am a:
- ◯ Physician (MD, DO, MBBS)
- ◯ Nurse Practitioner (CNP or Physician assistant (PA)

28) Approximately how many years of post-graduate clinical experience (with at least 20% of your time seeing patients) do you have?
- ◯ 1- 5 years
- ◯ 6-10 years
- ◯ 10-15 years
- ◯ 20+ years

29) I have received the following training in palliative care (check all that apply):
- ☐ Formal palliative care fellowship (1 year or more)
- ☐ Formal palliative care rotation (1 month or more)
- ☐ Palliative care courses, continuing medical education lectures or conferences
- ☐ No training
